# Autocrine CSF-1R signaling drives mesothelioma chemoresistance via AKT activation

**DOI:** 10.1038/cddis.2014.136

**Published:** 2014-04-10

**Authors:** M Cioce, C Canino, C Goparaju, H Yang, M Carbone, H I Pass

**Affiliations:** 1 Division of Thoracic Surgery, Department of Cardiothoracic Surgery, Langone Medical Center, New York University, New York, NY, USA; 2 University of Hawaii Cancer Center, John A Burns School of Medicine, University of Hawaii, Honolulu, HA, USA

**Keywords:** M-CSF, chemoresistance, EMT, mesothelioma, transformation

## Abstract

Clinical management of malignant pleural mesothelioma (MPM) is very challenging because of the uncommon resistance of this tumor to chemotherapy. We report here increased expression of macrophage colony-stimulating-factor-1-receptor (M-CSF/CSF-1R) mRNA in mesothelioma *versus* normal tissue specimens and demonstrate that CSF-1R expression identifies chemoresistant cells of mesothelial nature in both primary cultures and mesothelioma cell lines. By using RNAi or ligand trapping, we demonstrate that the chemoresistance properties of those cells depend on autocrine CSF-1R signaling. At the single-cell level, the isolated CSF-1R^pos^ cells exhibit a complex repertoire of pluripotency, epithelial–mesenchymal transition and detoxifying factors, which define a clonogenic, chemoresistant, precursor-like cell sub-population. The simple activation of CSF-1R in untransformed mesothelial cells is sufficient to confer clonogenicity and resistance to pemetrexed, hallmarks of mesothelioma. In addition, this induced a gene expression profile highly mimicking that observed in the MPM cells endogenously expressing the receptor and the ligands, suggesting that CSF-1R expression is mainly responsible for the phenotype of the identified cell sub-populations. The survival of CSF1R^pos^ cells requires active AKT (v-akt murine thymoma viral oncogene homolog 1) signaling, which contributed to increased levels of nuclear, transcriptionally competent *β*-catenin. Inhibition of AKT reduced the transcriptional activity of *β*-catenin-dependent reporters and sensitized the cells to senescence-induced clonogenic death after pemetrexed treatment. This work expands what is known on the non-macrophage functions of CSF-1R and its role in solid tumors, and suggests that CSF-1R signaling may have a critical pathogenic role in a prototypical, inflammation-related cancer such as MPM and therefore may represent a promising target for therapeutic intervention.

The colony-stimulating-factor receptor-1 (CSF-1R) is a type III receptor tyrosine kinase primarily reported as responsible for the proliferation, differentiation and survival of the monocyte–macrophage cell lineage, as well as their recruitment.^1^ The two known ligands of CSF-1R are CSF-1 and the structurally unrelated sibling IL-34 (interleukin-34). Both CSF-1 and IL-34 function through non-competitive binding to CSF-1R by triggering auto- and transphosphorylation of tyrosine residues in the cytoplasmic domain of the receptor,^2^ which results in the activation of both phosphatidylinositol 3-kinase (PI3K)-dependent and RAS-activated protein kinase-dependent pathways.^3^ CSF-1R may function in cancer cells as an oncogene^4^ by exploiting Src-dependent and -independent signaling modalities.^5, 6^ *In vivo*, elevated expression of CSF-1R has been described in tumors of epithelial origin, such as ovarian, endometrial, breast, prostate, renal and ovarian carcinomas, and has also been found to correlate in some cases with adverse prognosis.^7, 8, 9, 10, 11^ Studies in breast cancer and renal cell carcinoma suggest that autocrine signaling of CSF-1R may be relevant *in vivo* as well and modulated by oncogenic signaling (i.e. transforming growth factor-*β*1 (TGF*β*1) and EGF, respectively).^11, 12^

Malignant pleural mesothelioma (MPM) is a neoplastic disease of the pleura with a clear pathogenetic link to chronic inflammation, in most cases due to asbestos exposure.^13^ A clinically silent progression and an unusually high resistance to therapy make the prognosis of mesothelioma patients almost invariably poor and survival has changed very little in the past 20 years.^14^ MPM is a clinically relevant model to study the relationships between chronic inflammation and chemoresistance, as asbestos exposure of pleural mesothelial cells induces sustained nuclear factor-*κ*B (NF-*κ*B) activation^15^ following activation of high-mobility group box 1 signaling.^16, 17^ Here we show, for the first time, that expression of the CSF-1R identifies a specific cell sub-population exhibiting pluripotency and chemoresistance in both primary MPM cultures and cell lines. A specific gene expression profile and autocrine activation of the CSF-1R signaling characterize the functional uniqueness of these cells, which exhibit increased NF-*κ*B, signal transducer and activator of transcription 3 (STAT3) and AKT (v-akt murine thymoma viral oncogene homolog 1) activation. AKT activation following the forced activation of CSF-1R signaling in untransformed mesothelial cells confers accelerated growth, clonogenicity and resistance to pemetrexed, and leads to increased nuclear levels of functional *β*-catenin. This phenotype is reversed by inhibition of AKT. This study expands what is known on the function of CSF-1R in non-hematopoietic neoplasms and suggests that interference with the signaling of the CSF-1R^pos^ cells may overcome the chemoresistance of MPM.

## Results

### Mesothelioma CSF-1R^pos^ cells exist *in vivo*

**Figure 1:**
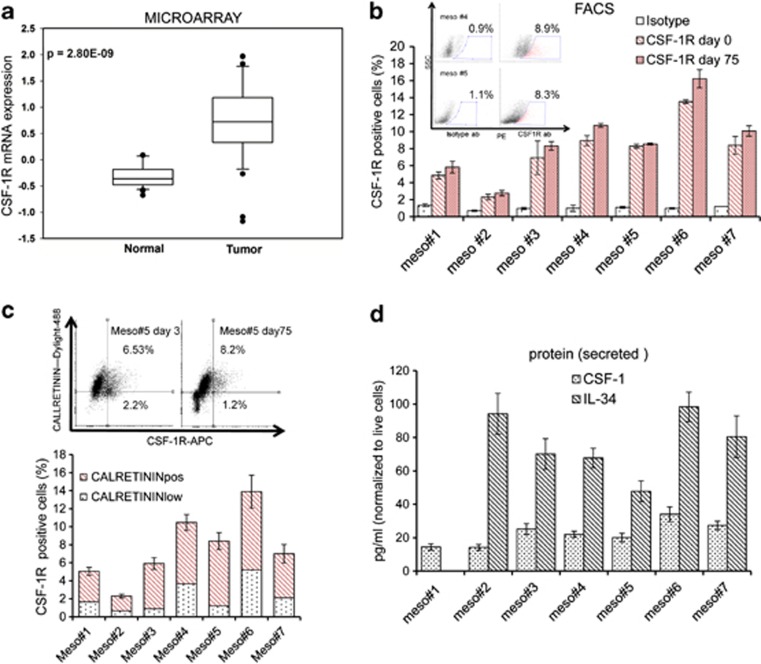
Mesothelioma CSF-1R^pos^ cells exist *in vivo*. (**a**) Box plot showing the levels of CSF-1R mRNA in matched peritoneal *versus* mesothelioma samples from Affymetrix microarray. (**b**, inset) Representative FACS dot plots of two mesothelioma primary cultures stained with anti-CSF-1R antibody (right) and an isotype-matched antibody (left) (as a control) at day 75. Gated positive cells are in red. (Main) Histograms showing the percentage of CSF-1R^pos^ cells in seven mesothelioma primary cultures stained with an anti-CSF-1R antibody (right) or the isotype-matched antibody (left) at the indicated times after harvesting. Note that no decrease in the number of CSF-1R^pos^ cells was observed after long-term culturing of the primary specimens. (**c**) The primary CSF-1R^pos^ cells are of mesothelial origin. (Upper panel) Representative FACS dot plots of the meso no. 5 primary cells assayed for CSF-1R and CALRETININ expression at days 3 and 75 after seeding, respectively. (Lower panel) Histograms showing the average percentage of Calretinin^pos^ and low/neg cells in the CSF-1R^pos^ fraction of the same mesothelioma primary cultures at 60–90 days after seeding (average time: 70 days). (**d**) Histograms show the levels of CSF-1 and IL-34 in the conditioned media of the indicated primary MPM cultures, as assessed by ELISA assay at day 60 of culture. Fresh cell growth medium was used as a background control. Bars indicate mean values±S.E.M. of at least two independent experiments

To investigate whether CSF-1R is expressed in mesothelioma tissues and if its levels are correlated to the tumor state, we performed a microarray expression analysis of 34 matched pair samples of normal peritoneal tissue *versus* mesotheliomas (Figure 1a). This revealed an increased mRNA expression of CSF-1R in the mesothelioma tissue as opposed to the mesothelial tissue (*P*<0.001). This finding did not exclude that tumor-associated macrophages or bone marrow-derived cells may contribute the observed higher CSF-1R expression in the tumor tissue. Therefore, we developed mesothelioma primary cultures (*n*=7) from pleural effusions and solid specimens of MPM patients. Fluorescence-activated cell sorting (FACS) staining revealed that all the MPM specimens contained CSF-1R^pos^ cells shortly after harvesting (*t*=2–18 h). Staining of the same samples after long-term culturing (2.2±1.1 months: average length of the culture) revealed that the percentage of CSF-1R^pos^ cells was almost unchanged or slightly increased in time (Figure 1b). Costaining with anti-calretinin antibodies indicated that a relatively large fraction of the CSF-1R^pos^ cells within the primary cell cultures expressed the mesothelioma-specific antigen calretinin,^18^ thus confirming the mesothelial nature of the CSF-1R^pos^ cells (Figure 1c). Finally, we assessed the levels of CSF-1 and IL-34, the known ligands for CSF-1R, in the conditioned medium of the primary cultures (*t*=45–60). Enzyme-linked immunosorbent assay (ELISA) assay revealed that both CSF-1R ligands were represented in the medium of the primary cultures (in 7/7 cultures and 6/7 cultures for CSF-1 and IL-34, respectively) (Figure 1d). The absence, at day 60 of culture, of detectable, surviving hematopoietic cells (lymphocytes, monocytes, eosinophils and neutrophils), as judged by the absence of CD3-, CD14-, CD16-, CD19-, CD20-, CD56- and CD45-positive cells within the CSF-1R^pos^ cell fraction (Supplementary Figure 1), suggested that mesothelial CSF-1R^pos^ cells could be responsible for the production of the CSF-1R ligands.

### MPM cell lines secrete CSF-1 and IL-34 and express functional CSF-1R

**Figure 2:**
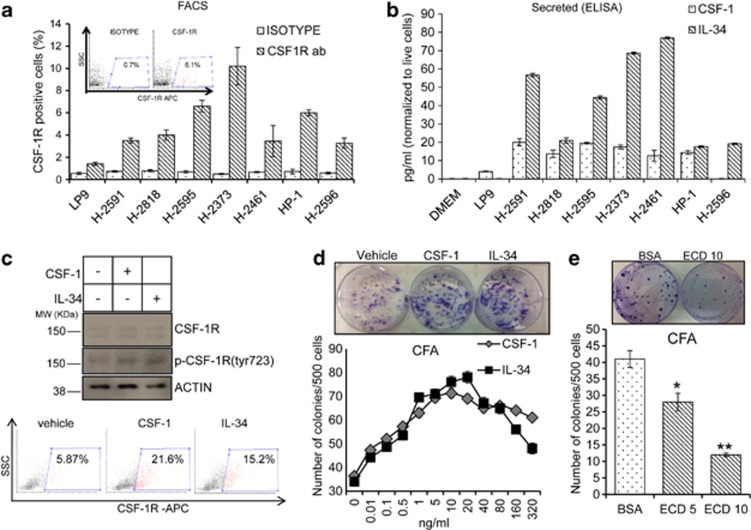
MPM cell lines secrete CSF-1 and IL-34 and express functional CSF-1R. (**a**, inset) Representative FACS dot plots of H-2595 cells stained with an anti-CSF-1R antibody (right) and an isotype-matched antibody (left) (as a control). Percentages of positive cells (red) are indicated. (Main) Histograms showing the percentage of CSF-1R^pos^ cells in multiple MPM cell lines, as assessed by FACS. For each cell line, the values of the isotype-matched control antibody are reported. Mean values±S.E.M of triplicate experiments are reported. (**b**) ELISA assay. Histograms show the levels of CSF-1 and IL-34 in the conditioned media of the same MPM cell lines. Fresh cell growth medium was used as a control. Bars indicate mean values±S.E.M. of at least two independent experiments. (**c**, upper panel) Representative western blotting of whole-cell lysates from starved H-2595 cells treated as indicated for 5 min at 37 °C and stained with an anti-CSF-1R, phospho-CSF1R(Tyr723) and actin (as a loading control). (Lower panel) FACS dot plots of the same cells as from upper panel treated as indicated for 48 h. Gates were drawn based on the background staining of isotype-matched antibodies. (**d**) Colony-forming assay (CFA). H-2595 cells were exposed to vehicle, CSF-1 and IL-34 for 16 h and then seeded at clonal density in six-well tissue culture dishes. (Upper panel) Representative micrographs of crystal violet-stained tissue culture dishes at 9 days after seeding. (Lower panel) Graph illustrating the number of colonies formed after the treatment of H2595 cells with different concentrations of ligands. (**e**) Histograms showing the average number of colonies (CFA) formed by H-2595 cells treated with the indicated concentrations of ECD (mg/ml) for 16 h before seeding at clonal densities. (Inset) Representative micrographs of the colonies formed by the BSA- and ECD-treated H-2595 cells. *P<0.05; **P<0.01 *versus* the corresponding controls (vehicle-treated cells)

To obtain a suitable experimental system to study the CSF-1R function in mesothelioma cells, we analyzed the expression of CSF-1R and its ligands CSF-1 and IL-34 in a panel of mesothelioma cell lines and an untransformed mesothelial cell line immortalized by h-TERT (LP9) (Figure 2). FACS analysis showed that all the mesothelioma cell lines contained a small sub-population of CSF-1R^pos^ cells (range 2–13%). A small percentage of LP9 cells exhibiting the expression of CSF-1R (<1.5%) was also present in the mesothelial cells (LP9) (Figure 2a). Next, ELISA assay revealed that 7/7 mesothelioma cell lines secreted IL-34 and 6/7 MPM cell lines secreted CSF-1, with the levels of IL-34 being generally higher than those of CSF-1 (Figure 2b). No detectable IL-34 and very little CSF-1 was produced by the untransformed mesothelial LP9 cells (Figure 2b). Thus, mesothelioma cell lines expressed all the components of the CSF-1R signaling module, implying active signaling in those cells. To verify this, we treated H-2595 cells with vehicle (phosphate-buffered saline (PBS)), CSF-1 (25 ng/ml) or IL-34 (25 ng/ml). This revealed increased CSF-1R autophosphorylation, as assessed by western blotting with phospho-CSF-1R (Tyr723) antibodies (Figure 2c, upper panel) in the cytokine-treated cells. This correlated with a strong increase of the CSF-1R^pos^ cells in the ligand-treated samples, as assessed by FACS (Figure 2c, lower panel). Next, we observed a dose-dependent increase of the formed colonies in the CSF-1- and IL-34-treated cells, which matched the increase of CSF-1R^pos^ cells observed in Figure 2c (Figure 2d, upper and lower panel). To prove that the increased clonogenicity was specifically due to CSF-1 and IL-34 binding, we treated H-2595 cells with a truncated CSF-1R containing the extracellular domain (ECD), shown to bind to and sequester both CSF-1 and IL-34.^19^ This affected the clonogenicity of the cells in a dose-dependent manner. No effect of the control (bovine serum albumin (BSA)) treatment was observed (Figure 2e and inset). We performed identical observations with the H-2373 cells (Supplementary Figures 2A–C).

### CSF-1R signaling mediates resistance to pemetrexed of MPM cell lines and primary samples

**Figure 3:**
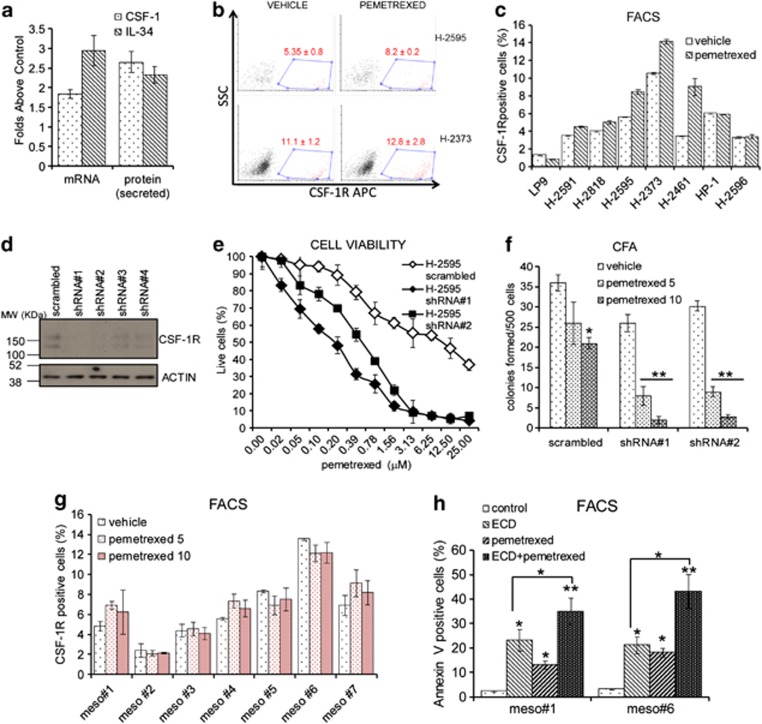
CSF-1R signaling mediates resistance to pemetrexed. (**a**) Pemetrexed treatment increases CSF-1 and IL-34 production. Histograms showing the relative increase of CSF-1 (white bars) and IL-34 (gray bars) mRNAs and of their secreted products in pemetrexed-treated H-2595 cells, expressed as folds above the control (vehicle). (**b** and **c**) CSF-1R^pos^ cells survive pemetrexed treatment. Representative FACS dot plots of H-2595 (upper panel) and H-2373 (lower panel) cells treated with vehicle and pemetrexed for 96 h and stained with an anti-CSF-1R antibody. The dot plots relative to the isotype-matched antibody-stained samples (control) are available in Supplementary Figure 6. Percentage of positive cells is shown. Mean values±S.E.M. of triplicate experiments are reported. (**c**) Histograms showing the percentage of CSF-1R^pos^ cells in vehicle (white bars)- and pemetrexed-treated (gray bars) samples from multiple mesothelioma cell lines treated as in (**b**). (**d**–**f**) RNAi-mediated downregulation of CSF-1R sensitizes H-2595 cells to pemetrexed. (**d**) Representative western blotting of whole-cell lysates from H-2595 cells transduced with a scrambled- and four CSF-1R-targeting short hairpin RNAs (shRNAs). Staining with anti-CSF-1R antibody revealed a deep downregulation of the CSF-1R protein levels, mostly with the shRNA nos. 1 and 2. Actin staining was used as a loading control. (**e**) Viability assay. H-2595 cells, expressing a scrambled- or two CSF-1R-targeting shRNAs, were treated with pemetrexed at the indicated doses and viable cells counted by the trypan blue exclusion assay at 96 h. (**f**) Clonogenic assay. The same cells as from (**e**) were pulse treated (16 h) with two increasing dosages of pemetrexed before being seeded at clonal densities. Histograms showing the average colony count at 9 days after seeding. Values indicate mean values±S.E.M. of at least duplicate experiments. *P<0.05; **P<0.01 *versus* the corresponding controls (vehicle-treated cells). (**g**) Primary CSF-1R^pos^ cells survive pemetrexed treatment. Histograms showing the percentage of CSF-1R^pos^ cells in seven mesothelioma primary cultures treated with vehicle or increasing doses of pemetrexed for 96 h and stained with an anti-CSF-1R antibody (right) or the isotype-matched antibody (left). (**h**) Primary CSF-1R^pos^ cells are sensitive to the inhibition of CSF-1R signaling. Histograms showing the percentage of CSF-1R^pos^ cells in two representative mesothelioma primary cultures treated with the CSF-1R decoy (ECD) alone or in combination with pemetrexed for 96 h and stained with annexin-V. Bars indicate mean values±S.E.M. of triplicate experiments. *P<0.05; **P<0.01 *versus* the corresponding controls (vehicle or as indicated by the connectors)

The previous observations suggested that active CSF-1R signaling may transduce prosurvival signals (Figure 3). Therefore, we investigated the involvement of the CSF-1R/CSF-1/IL-34 system in mediating the resistance of the mesothelioma cells to pemetrexed. First, we observed a significant increase of the CSF-1 and IL-34 mRNA in the cells treated with pemetrexed for 48 h, which matched the increased secretion of the two cytokines in the medium (Figure 3a). Next, FACS staining revealed that the CSF-1R^pos^ cells survived pemetrexed treatment, with no decrease or increase of the receptor-positive cells in the surviving cell fraction (Figures 3b and c). This was not observed in the pemetrexed-treated mesothelial LP9 cells (Figure 3c) (*P*<0.05). Next, we transduced H-2595 and H-2373 cells with two independent CSF-1R-targeting shRNAs (out of four tested), which largely reduced the expression of CSF1R, as demonstrated by western blotting (Figure 3d). CSF-1R downregulation affected the viability of both control (vehicle)- and pemetrexed-treated cells (Figure 3e and Supplementary Figure 2D). A similar effect was observed on the clonogenicity of the same cells either when treated with vehicle or with increasing concentrations of pemetrexed (Figure 3f and Supplementary Figure 2E). Although CSF-1R downregulation affected the viability and clonogenicity of both control (vehicle)- and pemetrexed-treated cells, its action on the pemetrexed-treated cells was much stronger, consistent with a chemosensitizing effect. To broaden the relevance of these findings, we assessed whether CSF-1R^pos^ mesothelioma cells in primary cultures would exhibit similar behavior. When challenged with pemetrexed, all the primary cultures (*n*=7) exhibited surviving CSF-1R^pos^ cells, whose number did not significantly change upon treatment (Figure 3g). Viability of purified CSF1R^pos^ cells from two representative primary cultures was affected by treatment with the CSF-1R decoy (ECD) and further reduced by pemetrexed treatment (Figure 3h). These observations suggest that, like in the cell lines, the CSF-1R expression in primary MPM specimens identifies a chemoresistant cell population sensitive to CSF-1R inhibition, emphasizing the potential for CSF-1R signaling to impinge on the chemoresistance of mesothelioma cells.

### FACS-sorted CSF-1R^pos^ cells exhibit a distinct gene expression profile

**Figure 4:**
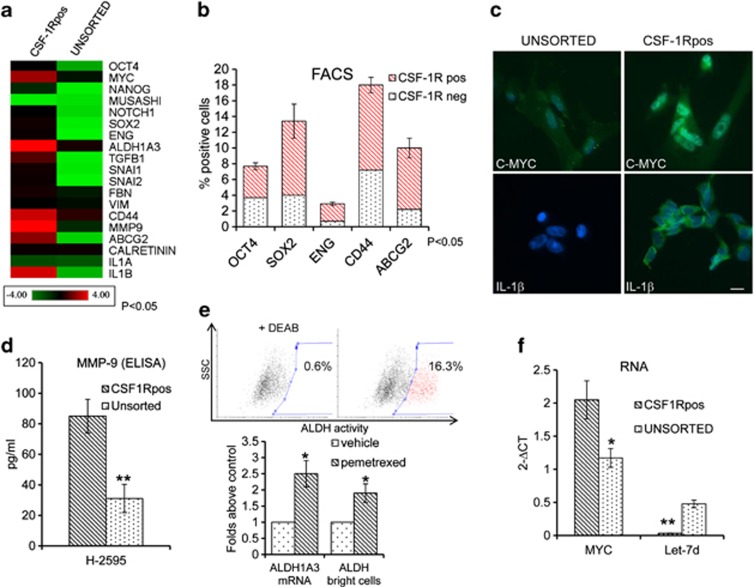
CSF-1R^pos^ cells are enriched for the expression of a complex set of pluripotency, EMT and chemoresistance factors. (**a**) Heat map illustrating the mRNA levels of the indicated factors in FACS-sorted CSF-1R*pos* as opposed to the unsorted parental cell population. Gene expression was assessed by quantitative PCR (average log 2 of the absolute intensity values of duplicate experiments has been used for the heat map). (**b**) Histograms showing the relative percentages of the CSF-1R^pos^ and CSF-1R^neg^ cells within the OCT4-, SOX2-, ENG-, CD44- and ABCG2-positive cell sub-populations, as assessed by FACS analysis of double-stained, unsorted cells. Values indicate mean values±S.E.M. of triplicate experiments. (**c**) Representative indirect IF micrographs of unsorted (left panels) and CSF-1R-purifed (right panels) cells stained with the indicated antibodies after fixing/permeabilization (3 days after sorting). Cell nuclei were stained with DAPI. (Upper panel) Anti-c-MYC/DAPI staining. (Lower panels) Anti-IL-1*β*/DAPI staining. Merged images were shown. Scale bar, 20 *μ*M. (**d**) CSF-1R^pos^ cells exhibit high MMP9 levels. Histograms showing the levels of MMP-9 protein in the conditioned medium of the indicated cell populations, as detected by ELISA (3 days after sorting). (**e**) CSF-1R^pos^ cells exhibit inducible ALDH1A3 mRNA and ALDH activity. (Upper panel) Representative FACS dot plots of purified CSF-1R^pos^ cells treated with an ALDH fluorescent substrate in the presence (left panel) or absence (right) of a specific ALDH inhibitor (DEAB). (Lower panel) Histograms showing the relative levels of ALDH1A3 mRNA and the percentage of ALDH(bright) cells in CSF-1R^pos^ cells treated with vehicle (white bars) or pemetrexed (gray bars), assessed by qRT-PCR and FACS, respectively. (**f**) CSF-1R^pos^ cells exhibit high c-MYC mRNA and low let-7d miRNA levels. Histograms showing the average normalized intensity values of c-MYC mRNA and let-7d miRNA in CSF-1R^pos^ cells as compared with their unsorted counterparts, as assessed by qPCR. Bars indicate mean values±S.E.M. of triplicate experiments. *P<0.05; **P<0.01 *versus* the corresponding controls (unsorted cells)

We investigated whether the repertoire of gene expression exhibited by the CSF-1R^pos^ cells could explain their properties (Figure 4). In detail, we evaluated whether FACS-purified, CSF-1R^pos^ H-2595 cells would differ from their unsorted counterparts with regard to the expression of pluripotency markers and chemoresistance-associated genes. Quantitative reverse-transcription PCR (qRT-PCR) analysis revealed enrichment of the CSF-1R^pos^ cells for the expression of *NANOG*, *OCT4*, *SOX2*, *ENDOGLIN*, *c-MYC* and NOTCH1 mRNAs. We also found very high levels of ATP-binding cassette transporters, subfamily G, member 2 (*ABCG2*) mRNA, a membrane drug transporter whose increased expression confers chemoresistant features.^20, 21^ Epithelial–mesenchymal transition (EMT) genes, such as *VIMENTIN*, *FBN*, matrix metalloproteinase-9 (*MMP-9*) and *CD44*, and chief EMT modulators, such as *SNAI1*, *SNAI2* and *TGF*-*β*1, were also enriched in CSF-1R^pos^ cells. We also found that the CSF-1R^pos^ cells were enriched for IL-1*β*, shown to modulate CSF-1 and IL-34, in a NF-*κ*B-dependent manner^22^ (Figure 4a, heat map). We used several approaches to validate some of these findings: FACS analysis of double-stained H-2595 and H-2373 cells confirmed the relative enrichment of CSF-1R^pos^ cells for the expression of *CD44*, *OCT4*, *SOX2*, *ENDOGLIN* and *ABCG2* in unsorted cell populations (Figure 4b and Supplementary Figure 3A, respectively). Additionally, we confirmed higher levels of *c-MYC* and *IL-1β* in the CSF-1R^pos^ cells by indirect immunofluorescence (IF) (Figure 4c). ELISA assays on conditioned media from the sorted cells confirmed the presence of high levels of MMP-9 (Figure 4d and Supplementary Figure 3B). In addition, according to the enrichment of the sorted cells for aldehyde-dehydrogenase 1A3 (*ALDH1A3*) mRNA (Figure 4a), we evaluated the presence of ALDH activity in the CSF-1R^pos^ cells. Briefly, we analyzed the accumulation of a fluorescent ALDH substrate into the cells, by using those treated with a specific ALDH inhibitor (DEAB (diethylaminobenzaldehyde)) as a background control (ALDEFLUOR Kit, STEMCELL Technologies Inc., Vancouver, BC, Canada). This showed that the CSF-1R^pos^ cells were endowed with ALDH activity (Figure 4e, upper panel). ALDH is a detoxifying enzyme whose function has been linked to cancer chemoresistance.^23^ Indeed, both *ALDH1A3* mRNA and ALDH activity were significantly increased by pemetrexed treatment in the CSF-1R^pos^ cells, as shown by qRT-PCR and FACS, respectively (Figure 4e, lower panel and Supplementary Figure 3C), thus further supporting the chemoresistant properties of this cell sub-population. Next, we found that the high levels of c-MYC in the CSF-1R^pos^ cells inversely correlated with those of the mature microRNA (miRNA) let-7d (Figure 4f and Supplementary Figure 3D), a typical feature of undifferentiated cells, in both H-2595 and H-2373 CSF1R^pos^ cells.^24^

### CSF-1R^pos^ cells rely on autocrine signaling to AKT for their survival

**Figure 5:**
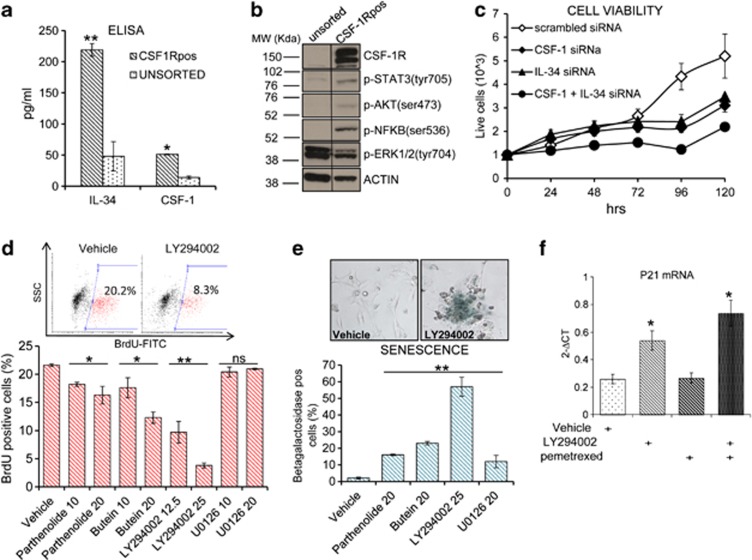
CSF-1R^pos^ cells rely on autocrine-, CSF-1- and IL-34-mediated activation of AKT. (**a**) ELISA assay. Histograms showing the average quantity of CSF-1 and IL-34 in the medium conditioned by CSF-1R^pos^ cells at day 3 after sorting. (**b**) Representative western blotting of whole-cell lysates from sorted CSF-1R^pos^ and the unsorted parental cells stained with the indicated antibodies. Note that reported are two regions of the same filter used for the western blotting. (**c**) CSF-1 and IL-34 production support survival of the CSF-1R^pos^ cells. Cell growth of CSF-1-, IL-34-, CSF-1+IL-34 RNAi-transfected CSF-1R^pos^ cells at day 3 after sorting, as assessed by trypan blue exclusion staining. (**d**) STAT3, NF-κB and AKT inhibitors affect the growth of CSF-1R^pos^ cells. (Upper panel) Representative FACS dot plots of purified CSF-1R^pos^ cells treated with LY294002, a PI3K/AKT inhibitor and pulsed with bromodeoxyuridine 48 h later. (Lower panel) Histograms showing the percentage of Brdu-positive cells in purified CSF-1R^pos^ cells treated with the indicated compounds for 48 h. (**e**) STAT3, NF-κB and AKT inhibitors trigger senescence of CSF-1R^pos^ cells. SA-*β*-gal assay. (Upper panel) Representative micrograph of CSF-1R^pos^ cells treated with vehicle or LY294002 (12.5 mm) for 7 days and stained for *β*-gal activity. Medium containing the inhibitors was replaced every 48 h. (**f**) PI3K/AKT inhibition upregulates p21 mRNA levels in purified CSF-1R^pos^ cells. Histograms showing the levels of p21mRNA in the CSF-1R^pos^ cells treated as indicated for 24 h. Data expressed as absolute intensity values normalized for a housekeeping gene (*PPIA*). Bars indicate mean values±S.E.M. of triplicate experiments. *P<0.05; **P<0.01; NS: not significant *versus* the corresponding controls (vehicle or unsorted cells)

Both CSF-1 and IL-34 mRNAs were expressed by the (Figure 5) purified CSF-1R^pos^ cells and secreted in the medium, thereby demonstrating the existence of autocrine production of the ligands (Figure 5a). This was functionally relevant, as western blotting revealed high enrichment of the purified CSF-1R^pos^ cells for phospho-STAT3 (Tyr705), phospho-NF-*κ*B (p-p65-Ser536) and phospho-AKT (Ser473). No differences of the phospho-extracellular signal-regulated kinase 1/2 (ERK1/2) (Tyr704) signal were observed between unsorted and CSF-1R^pos^ cells (Figure 5b). Next, we investigated the relevance of the autocrine CSF-1 and IL-34 production for the survival of the CSF-1R^pos^ cells. We treated the purified cells with scrambled-, CSF-1- or IL-34-targeting small interfering RNAs (siRNAs) (20 pM, 48 h), which strongly reduced the levels of CSF-1 and IL-34 mRNAs (Supplementary Figures 4A and B). Cell proliferation assays showed that the depletion of either of the ligands by siRNA affected the growth of the CSF-1R^pos^ cells, with the strongest effect following the simultaneous downregulation of both ligands (Figure 5c). Next, we investigated which of the signaling events, downstream to CSF-1R activation, could promote the survival of the CSF-1R^pos^ cells, thereby potentially affecting their chemoresistance. We evaluated the effect of two naturally occurring, polyfunctional NF-*κ*B/STAT3 inhibitors, namely parthenolide and butein (2′,3,4,4′-tetrahydroxychalcone), and two more specific AKT and ERK small-molecule inhibitors, namely LY294002 (PI3K/AKT) and U0126 (MEK1/2–ERK1/2). These compounds were chosen for their ability to counteract the chemoresistance of malignant mesothelioma and other tumor models.^25, 26, 27, 28^ FACS analysis of the purified, bromodeoxyuridine (BrdU)-labeled CSF-1R^pos^ cells (from H-2595 and H-2373 cells) revealed that three of the tested compounds were capable of significantly decreasing the growth of the H-2595- and H-2373-derived CSF-1R^pos^ cells, with the ERK inhibitor showing no effect and the AKT inhibitor the strongest one (Figure 5d and Supplementary Figure 4C). Staining of the treated cells for SA-*β*-galactosidase (*β*-gal) activity showed that the lack of BrdU incorporation was mainly due to senescence, with the LY294002 treatment eliciting the highest number of senescent cells (Figure 4e). This correlated with the upregulation of p21 in the LY294002 CSF-1R^pos^-treated cells, which was further increased in the pemetrexed-treated samples (Figure 5f). Thus, CSF-1R activation may trigger prosurvival stimuli by reducing spontaneous senescence mainly via AKT activation.

### CSF-1R transforms mesothelial cells

**Figure 6:**
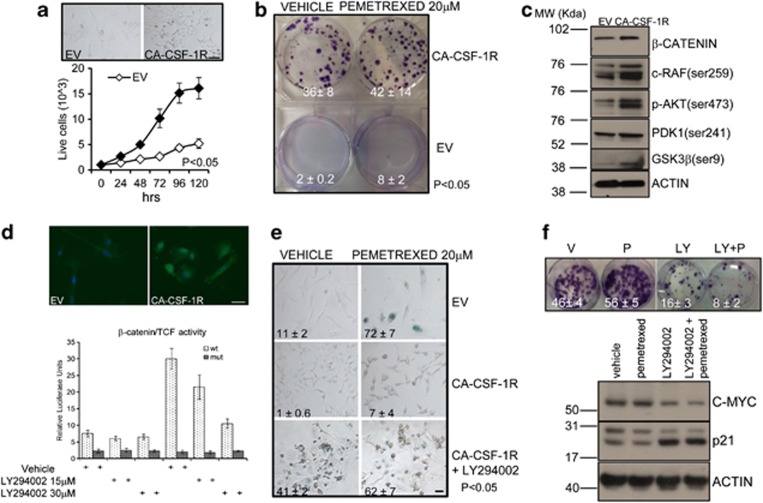
CSF-1R transforms mesothelial cells. Untransformed mesothelial cells (LP9) were transduced with an EV (left) and a vector encoding for a ligand-independent, constitutively active CSF-1R (CA-CSF-1R, right) and the resulting cell sublines assayed for their transformation status. (**a**, upper panel) Representative micrographs of EV- and CA-CSF-1R-expressing cells, respectively. Note the morphological changes induced by CA-CSF-1R expression. Scale bar, 100 *μ*M. (Lower panel) Proliferation assay. EV- and CA-CSF1R-LP9 cell number was assessed by manual cell counting at the indicated times. (**b**) Clonogenic assay. Representative micrographs of crystal violet-stained colonies from EV- and CA-CSF-1R-LP9 cells pulsed with vehicle or pemetrexed and seeded at clonal density. The average percentage±S.E.M. of the formed colonies from triplicate experiments is indicated in each quadrant. (**c**) Representative western blotting of whole-cell lysates from logarithmically growing CA-CSF-1R- and EV-LP9 cells. Staining with the indicated antibodies indicated high enrichment of the CA-CSF-1R-LP9 cells for phosphorylated AKT, RAF, PDK1 and GSK3*β*, suggestive of full activation of the AKT pathway. Additionally, higher levels of *β*-catenin were observed in the CA-CSF-1R-LP9 cells. Actin was used as an internal loading control. (**d**) Forced activation of CSF-1R in LP9 cells increases the levels of functional *β*-catenin complexes. (Upper panel) Indirect IF of EV- and CA-CSF-1R-LP9 cells stained for *β*-catenin. Nuclei were decorated with DAPI. Merged images were shown. Scale bar, 20 *μ*M. (Lower panel) Luciferase assay. Histograms indicate the levels of luciferase activity from EV- and CA-CSF-1R-LP9 cells transfected with vectors containing wild-type (wt) or mutated *β*-catenin/TCF-binding sites upstream of the luciferase cDNA. Values normalized to *Renilla* luciferase units. (**e**) CA-CSF-1R expression reduces the pemetrexed-induced senescence in an AKT-dependent manner. (Upper and middle panels) Representative micrographs of CA-CSF-1R and EV-LP9 treated with vehicle and pemetrexed for 5 days and assayed for the presence of *β*-gal-positive, senescent cells. (Lower panels) CA-CSF-1R-LP9 cells pretreated with LY294002(12.5 *μ*M) before being processed as indicated for the cells in the upper and middle panels. The percentages of *β*-gal-positive cells from two triplicate experiments are indicated in each quadrant. Scale bar, 100 *μ*M. (**f**) AKT inhibition reverses the resistance of CA-CSF-1R-expressing cells to pemetrexed. (Upper panel) Clonogenic assay. Representative micrographs of crystal violet-stained colonies from CA-CSF-1R-LP9 cells pulsed with vehicle or pemetrexed (20 *μ*M) with or without pretreatment with LY294002 (12.5 *μ*M). The average percentage±S.E.M. of the formed colonies from triplicate experiments is indicated in each quadrant. V=vehicle; P=pemetrexed; LY=LY294002. (Lower panel) AKT inhibition upregulates p21 and downregulates c-MYC in CA-CSF-1R-LP9 cells. Representative western blotting of whole-cell lysates from CA-CSF-1R and EV-LP9 cells stained with the indicated antibodies. Actin was used as a loading control. *P<0.05; **P<0.01 *versus* the corresponding controls

To understand whether the active CSF-1R signaling was sufficient to drive malignant properties in untransformed mesothelial cells, we expressed a ligand-independent, constitutively active mutant of CSF-1R (CA-CSF-1R: harboring a mutation in the ligand-binding domain and in the extracellular tail)^29, 30^ in the LP9 cells (Figure 6). The latter are characterized by slow growth, no clonogenicity and sensitivity to chemotherapy. We found that the expression of CA-CSF-1R triggered changes in cell morphology and growth kinetics by conferring to the LP9 cells, a epithelial, loosely adherent phenotype and a much faster growth rate (as compared with the empty-vector-infected ones: EV-LP9) (Figure 6a, upper and lower panels, respectively). qRT-PCR demonstrated that the gene expression changes in the CA-CSF-1R-expressing cells closely mimicked what was observed in the cells endogenously expressing CSF-1R (Supplementary Figure 5A). Western blotting confirmed the increased expression of SOX2 and MUSASHI-1 in the CA-CSF-1R-expressing cells (Supplementary Figure 5B). In addition, the CA-CSF-1R-expressing LP9 cells were clonogenic and resistant to pemetrexed treatment (Figure 6b). Based on the previous findings (Figure 5), we evaluated the AKT activation status in CA-CSF-1R- and EV-LP9. Western blotting revealed that, in addition to increased levels of serine- and threonine-phosphorylated AKT, the CA-CSF-1R-LP9 cells exhibited increased levels of phosphorylated PDK1(Ser241) and glycogen synthetase kinase-3*β* (GSK3*β*) (Ser9), and main upstream and downstream effectors of AKT signaling, respectively (Figure 6c). We also observed increased phosphorylated c-RAF (Ser259), which connects the mitogen-activated protein kinases (ERK1/2) activation to the AKT pathway^31^ (Figure 6c). Phosphorylation of GSK3*β* at serine 9 inactivates this kinase activity and causes stabilization and nuclear localization of *β*-catenin from the plasma membrane to the cytoplasm and the nucleus, where the latter acts as a transcription cofactor for developmentally regulated genes.^32^ According to this, western blotting revealed increased levels of *β*-catenin in the CA-CSF-1R-LP9 cells (Figure 6c). Additionally, indirect IF indicated increased nuclear localization of *β*-catenin in the CA-CSF-1R-LP9 cells as compared with their controls (Figure 6d, upper panel). To assess whether the nuclear-localized *β*-catenin was functional, we transfected *β*-catenin-responsive reporter vectors, containing wild-type or mutated TCF-binding sites, in both empty vector (EV) and CA-CSF-1R-expressing cells. This revealed a much higher transcriptional activity in the CA-CSF-1R-LP9 (Figure 6d, lower panel), in agreement with the increased levels and nuclear localization of the protein in those cells. Also, the *β*-catenin-dependent transcriptional activity was inhibited by treatment with the AKT inhibitor in a dose-dependent manner (Figure 6d, lower panel). This suggests that AKT activation contributed to the higher levels of functionally active *β*-catenin. Next, we evaluated the effect of inhibiting AKT on the response of EV- and CA-CSF-1R-LP9 to pemetrexed. SA-*β*-gal assay revealed that, while pemetrexed treatment strongly increased the number of senescent cells in the control EV-LP9 cells, no senescent cells were observed in the CA-CSF1R-expressing LP9 (Figure 6e, upper and middle panels). Notably, pretreatment of CA-CSF1R-LP9 cells with LY294002 strongly increased the number of senescent cells in both vehicle- and pemetrexed-treated cells (Figure 6f, lower panel) and only slightly increased the number of apoptotic cells (Supplementary Figure 5C). In line with the increase of senescent cells in the LY294002-treated samples, the clonogenic assays revealed that AKT inhibition reversed the resistance of CA-CSFR-expressing cells to pemetrexed (Figure 6f, upper panel). LY294002 treatment was accompanied by the upregulation of p21 and downregulation of c-MYC proteins in both vehicle- and pemetrexed-treated samples, as revealed by western blotting (Figure 6f, lower panel). This established a link between the expression of CA-CSF-1R expression, AKT activation and chemoresistance of the mesothelial cells.

## Discussion

Previous work has reported expression of the CSF-1R and its ligand CSF-1 in solid tissue tumors and cell lines, and in some cases this was shown to be prognostic or to correlate with the *in vitro* aggressiveness of the cell lines. However, no studies to date have isolated and characterized the CSF-1R-expressing cells and thereby exploring their biological properties at the single-cell level. Thus, we report a novel finding that CSF-1R expression identifies precursor-like, self-renewing mesothelioma cell sub-populations endowed with prominent protumorigenic properties, such as clonogenicity and chemoresistance. In addition, we showed that IL-34, structurally unrelated ligand for the CSF-1R, has a role in the maintainance of the CSF-1R^pos^ cell sub-populations. This potential oncogenic role of IL-34 is unprecedented.

The findings of this work may be clinically relevant. Indeed, we report increased expression of CSF-1R in mesothelioma patient samples as compared with matched normal peritoneal tissues (*n*=34). Besides, we showed that CSF-1R^pos^ cells exist and persist within long-term primary cultures (*n*=7) and are resistant to pemetrexed in a CSF-1R-dependent manner. The fact that the *ex vivo* cultures used in this study are virtually devoid of any hematopoietic component and that their strongly similar behavior to long-term established MPM cell lines (*n*=7) support what was observed here is mainly a property of stable, CSF-1R-expressing cells of mesothelial nature. Therefore, the experimental model adopted here potentially expands what is known on the tumor-supporting, macrophage-independent functions of the CSF-1R in an autocrine setting. The purified CSF-1R^pos^ cells exhibit enrichment for *SOX2*, *OCT4* and *c-MYC*, and express low miRNA-let-7d levels. In addition, those cells express *NOTCH1* and *ENDOGLIN*. Interestingly, Notch1 upregulation by the SV40 virus was causally linked to mesothelial cell immortalization.^33^ Endoglin promoted resistance of ovarian cancer cells to DNA-damaging agents *in vitro* and *in vivo*.^34^ In line with this, the expression of *ALDH1A3* mRNA and activity and the surface enrichment for drug-effluxing activities, such *ABCG2*, support the chemoresistant properties of the isolated cell sub-populations. Therefore, expression of all the above factors is likely to confer a precursor-like, chemoresistant status to the CSF-1R^pos^ cells.

In terms of signaling, we provide evidence that activation of AKT is central to the properties of the CSF-1R^pos^ cells, including survival and resistance to pemetrexed. In detail, AKT inhibition indeed counteracted the bypass of senescence induced by the expression of active CSF-1R in pemetrexed-treated mesothelial cells, thereby restoring their chemosensitivity. Interestingly, activation of *β*-catenin by CSF-1 was shown to promote survival and expression of c-MYC and CyclinD1 in bone-marrow-derived macrophages.^35^ Additionally, the increased functional competence of *β*-catenin upon expression of activated CSF-1R and its sensitivity to AKT inhibition shown here suggest that *β*-catenin-dependent transcriptional mechanisms may have a role in shaping the expression profile of the CSF-1R^pos^ cells. Our observations suggest that, in addition to that of AKT, activation of the STAT3 and NF-*κ*B pathways may take place in the CSF-1R^pos^ cells as well and contribute to the survival of the CSF-1R^pos^ cells. We have recently shown that functional interaction of STAT3 and NF-*κ*B impinges on mesothelioma resistance to pemetrexed by modulating EMT-associated genes and that butein abated the resistance of mesothelioma cells by decreasing the pSTAT3–NF-*κ*B interaction.^26^ This is interesting, given the functional and physical crosstalk between the AKT, STAT3 and NF-*κ*B pathways shown in other experimental systems (such as MYC-driven lymphomas).^36^ Thus, an intriguing degree of pathway interaction seems to sustain the expression of pluripotency and EMT factors, ultimately impinging on a chemoresistant phenotype.

An interesting aspect of this study is that interference with the survival of the CSF-1R^pos^ cells (typically representing <10% of the total live cells in unfractionated cultures) abated the resistance of both cell lines and primary samples to pemetrexed at a much greater extent than it would be expected by targeting only the CSF-1R^pos^ cells. It is possible that molecules secreted by the CSF-1R^pos^ cells may confer oncogenic signals to adjacent, non-CSF-1R^pos^ cells. With regard to this, the CSF-1R^pos^ cells have the potential to secrete TGF*β*1 and IL-1*β*, potent inducers of EMT, and EMT is a strong determinant of chemoresistance and a prognostic factor for mesothelioma.^37, 38^ Therefore, the CSF-1R^pos^ cells may act in this regard as tumor-supporting mesenchymal elements. The increase of IL-1*β* triggered by CSF-1R activation may also be relevant for the induction of CSF-1 and IL-34 expression, as shown in osteoblast studies.^22^

Thus, the CSF-1R^pos^ cells can be attractive targets *in vivo* as interference with their survival broadly affects the protumorigenic features of both mesothelioma primary cultures and cell lines. In this respect, our observations indicate that both CSF-1R and AKT inhibition may be clinically relevant tools to overcome mesothelioma chemoresistance. This may allow expansion of the poor therapeutic portfolio for such a fatal disease. It is also possible that the findings herein may be applied to other inflammation-related neoplasms, of which mesothelioma is a prototype.

## Materials and methods

### Cell lines and culture conditions

The human MPM cell lines H-2591, H-2818, H-2595, H-2373, H-2461, HP-1 and H-2596,^39^ and the immortalized mesothelial cell line, LP9,^40^ were cultured as monolayers in Advanced Dulbecco's modified Eagle's medium (DMEM)/F12+Glutamax (Invitrogen-Gibco, Carlsbad, CA, USA) supplemented with 5% non-heat inactivated fetal bovine serum (Life Technologies, Grand Island, NY, USA) at <37 °C and 5% CO_2_. Before treatment with the recombinant cytokines, cells were shortly (<16 h) cultured in the starvation medium (DMEM/F12+Glutamax) supplemented with 1% BSA-FAF (fatty acid-free; Sigma, St. Louis, MO, USA).

### Patient samples

mRNA from 34 snap-frozen samples (matched peritoneal *versus* MPM) was used for the initial screening of CSF-1R, and originated from operative specimens of one of the authors (HIP) from patients having cytoreductive surgery for MPM at the Karmanos Cancer Institute (Detroit, MI, USA), and from the NYU Langone Medical Center (New York, NY, USA). In all cases, harvesting of the tissue in a deidentified manner was approved by the respective Investigation Review Boards. Samples were profiled by using the GeneChip Human Gene 1.0 ST Arrays (Affymetrix, Santa Clara, CA, USA) according to the manufacturer's instructions.

### Primary MPM cultures

Primary MPM cultures (meso nos. 1–7) were generated according to our protocol available on http://www.bio-protocol.org/wenzhang.aspx?id=285, upon approval of the Investigation Review Board of NYU Langone Medical Center.

### Reagents

Recombinant human M-CSF receptor (Sino Biological, Daxing, China), recombinant human M-CSF (BioLegend, San Diego, CA, USA), recombinant human IL-34 (BioLegend), pemetrexed (Eli Lilly and Company, Indianapolis, IN, USA), LY294002 (Cell Signaling Technology, Danvers, MA, USA), U0126 (Cell Signaling Technology), parthenolide (Sigma) and butein (Santa Cruz Technology, Dallas, TX, USA) were dissolved according to the manufacturer's instructions.

### Retroviral transduction of H-2595, H-2373 and LP9 cells

The shRNA containing vectors HuSH pRFP-C-RS (Origene, Rockville, MD, USA) and MSCV-HumanCSF1R-IRES-GFP (a kind gift from Martin Roussel, Memphis, TN, USA) were transfected into 293T packaging cells (ATCC, Manassas, VA, USA) using Lipofectamine 2000 (Life Technologies) according to the manufacturer's instructions. After 48 h, the virus-containing supernatants were filtered (0.45 *μ*M) and used to infect H-2595, H-2373 or LP9 cells (three cycles of infection). Where possible (HuSH vectors), the infected cells were selected by puromycin (1 *μ*g/ml for 1 week).

### siRNAs

Silencer predesigned siRNA CSF-1 and siRNA IL-34 (Ambion-Life Technology, Foster City, CA, USA) were transfected into MPM cells using Lipofectamine 2000 (Invitrogen-Gibco) according to the manufacturer's instructions.

### Cell viability assay

Manual counting and trypan blue dye-exclusion assay were used to assess cell growth and the cell viability. When indicated, cell viability was also assessed by FACS analysis of SYTOX Dead Cell Stain-labeled cells (Life Technologies) according to the manufacturer's instructions.

### Clonogenic assays

MPM cell lines were grown to 70% confluence and pulse treated with the indicated drugs or transfected as indicated. After 16 h, cells were detached and seeded at 500–1500 cells per well into six-well dishes (Corning-Costar, Tewksbury, MA, USA) in drug-free media. Fresh media (25%) were added every 3 days. Colonies were stained with crystal violet (Sigma) and colonies (>50 cells) were counted after 7–14 days.

### ELISA

Cytokine quantification levels of CSF-1 (Abnova, Taipei City, Taiwan) and IL-34 (BioLegend) secreted in the conditioned media (72 h) were quantitated by ELISA.

### MMP-9 quantification

The levels of MMP-9 were quantified in the media conditioned by CSF-1R^pos^ cells and their unsorted counterparts by ELISA, according to the manufacturer's instructions (MMP-9 Human ELISA Kit; Life Technologies).

### RNA extraction

Total RNA was extracted using the RNAeasy minikit (Qiagen, Hilden, Germany).

### cDNA synthesis and gene expression

The first-strand cDNA was synthesized according to manufacturer's instructions (High Capacity RNA-to-cDNA Kit; Applied Biosystems, Foster City, CA, USA). Gene expression was measured by real-time PCR using the SYBR Green dye (Applied Biosytems) on a StepOne Instrument (Applied Biosytems). qPCR primers are reported in Supplementary Table 1. PPIA and 18S were used as an endogenous control.

### miRNA expression analysis

Let-7d was detected using a specific TaqMan miRNA assay (Applied Biosystems) according to the manufacturer's instructions on a StepOne Instrument (Applied Biosytems). RNU6B was used as an endogenous control.

### Lysate preparation and immunoblotting

Cells were lysed in buffer with 50 mM Tris-HCl (pH 8), with 1% NP-40 (Igepal AC-630), 150 mM NaCl and 5 mM EDTA. Extracts were centrifuged at 14 000 × *g* for 15 min to remove cell debris. Protein concentrations were determined by colorimetric assay (Bio-Rad, Hercules, CA, USA). Western blotting was performed using the primary antibodies as indicated in Supplementary Table 2. Actin was used as a loading control.

### Luciferase reporter assay

To evaluate *β*-catenin/TCF-4 transcriptional activity, we used a pair of luciferase reporter constructs, TOP-FLASH and FOP-FLASH (Upstate Biotechnology, Lake Placid, NY, USA). Plasmids of TOP-FLASH (with three repeats of the TCF-binding site) or FOP-FLASH (with three repeats of a mutated TCF-binding site) were transfected into cells treated with LY294002 according to the manufacturer's instructions. Luciferase activity was measured using the Dual-Luciferase Reporter Assay System (Promega, Madison, WI, USA), with *Renilla* luciferase activity as an internal control, 48 h after transfection.

### IF microscopy

Briefly, the cells were fixed and permeabilized in paraformaldehyde/methanol, aspecific binding blocked in PBS containing 1% BSA and labeled with the indicated antibodies. The secondary antibody was an Dylight Goat Anti-Rabbit IgG (Abcam, Cambridge, UK). The nuclei were visualized by 4′,6-diamidino-2-phenylindole (DAPI) staining. Merged images were shown.

### SA-*β*-gal staining

SA-*β*-gal staining was performed on adherent cells according to the manufacturer's instructions (Cell Signaling Technology).

### BrdU incorporation assay

Detection of BrdU incorporation was performed by ELISA (BrdU Cell Proliferation Assay Kit; Cell Signaling Technology) according to the manufacturer's instructions.

### Flow cytometry

Cells were detached by PBS 1 × /EDTA 2 mM, fixed with 4% PFA permeabilized (when needed) in 80% methanol (10 min on ice), washed two times with PBS 1 × and resuspended for antibody staining at 1 × 10^6^ cells per 100 *μ*l in PBS 1 × /BSA 1%. The list of the primary antibodies, isotype-matched controls and secondary antibodies is available in Supplementary Table 2. Gates were drawn to exclude >99% of aspecific staining (based on the isotype-stained samples). Data were acquired using a FACSCalibur Instrument (BD Biosciences, Franklin Lakes, NJ, USA) and analysis was performed by using the Flowing Software 2.0 (Cell Imaging Core, University of Turku, Turku, Finland).

### Apoptosis detection

Dead and apoptotic cells were measured by FACS analysis after propidium iodide/Annexin-V staining (Vybrant Apoptosis Assay Kit No. 3; Life Technologies) according to the manufacturer's instructions.

### ALDH activity assay

ALDH activity was detected by FACSCalibur instrument (BD Biosciences). ALDEFLUOR Kit (STEMCELL Technologies Inc.) was used according to the manufacturer's instructions. ALDH-positive cells were defined as the cells that displayed greater fluorescence compared with a control staining reaction containing the ALDH inhibitor, DEAB.

### Cell sorting

Cells were filtered through a 40 *μ*M mesh to obtain a single-cell suspension and were incubated with primary and secondary antibodies for 45 min on ice. Cell sorting was performed with a MoFlo cell sorter (Dako Cytomation, Fort Collins, CO, USA) or a BD ARIA II (BD Biosciences).

### Statistical analysis

The means of each data set were analyzed using a Student's *t*-test with a two-tailed distribution and assuming equal sample variance.

## Abbreviations

ALDH: aldehyde-dehydrogenase
DAPI: 4′,6-diamidino-2-phenylindole
DEAB: diethylaminobenzaldehyde
DMEM: Dulbecco's modified Eagle's medium
NF-*κ*B: nuclear factor-*κ*B
AKT: v-akt murine thymoma viral oncogene homolog 1
C-MYC: v-myc myelocytomatosis viral oncogene homolog
TGF-*β*: transforming growth factor-*β*
ABCG2: ATP-binding cassette transporters, subfamily G, member 2
PI3K: phosphatidylinositol 3-kinase
GSK3*β*: glycogen synthetase kinase-3*β*
STAT3: signal transducer and activator of transcription 3
MMP: matrix metalloproteinase
CD: cluster of differentiation
IL: interleukin
EMT: epithelial–mesenchymal transition
IF: immunofluorescence
siRNA: small interfering RNA
qRT-PCR: quantitative reverse-transcription PCR
miRNA: microRNA
FACS: fluorescence-activated cell sorting
ELISA: enzyme-linked immunosorbent assay
PBS: phosphate-buffered saline
EV: empty vector
