# Supplementary figures and images for: Autocrine CSF-1R signaling drives mesothelioma chemoresistance via AKT activation
Source: Cell Death Dis. 2014 Apr 10;5(4):e1167–. doi: 10.1038/cddis.2014.136 (PMC5424113; doi:10.1038/cddis.2014.136)

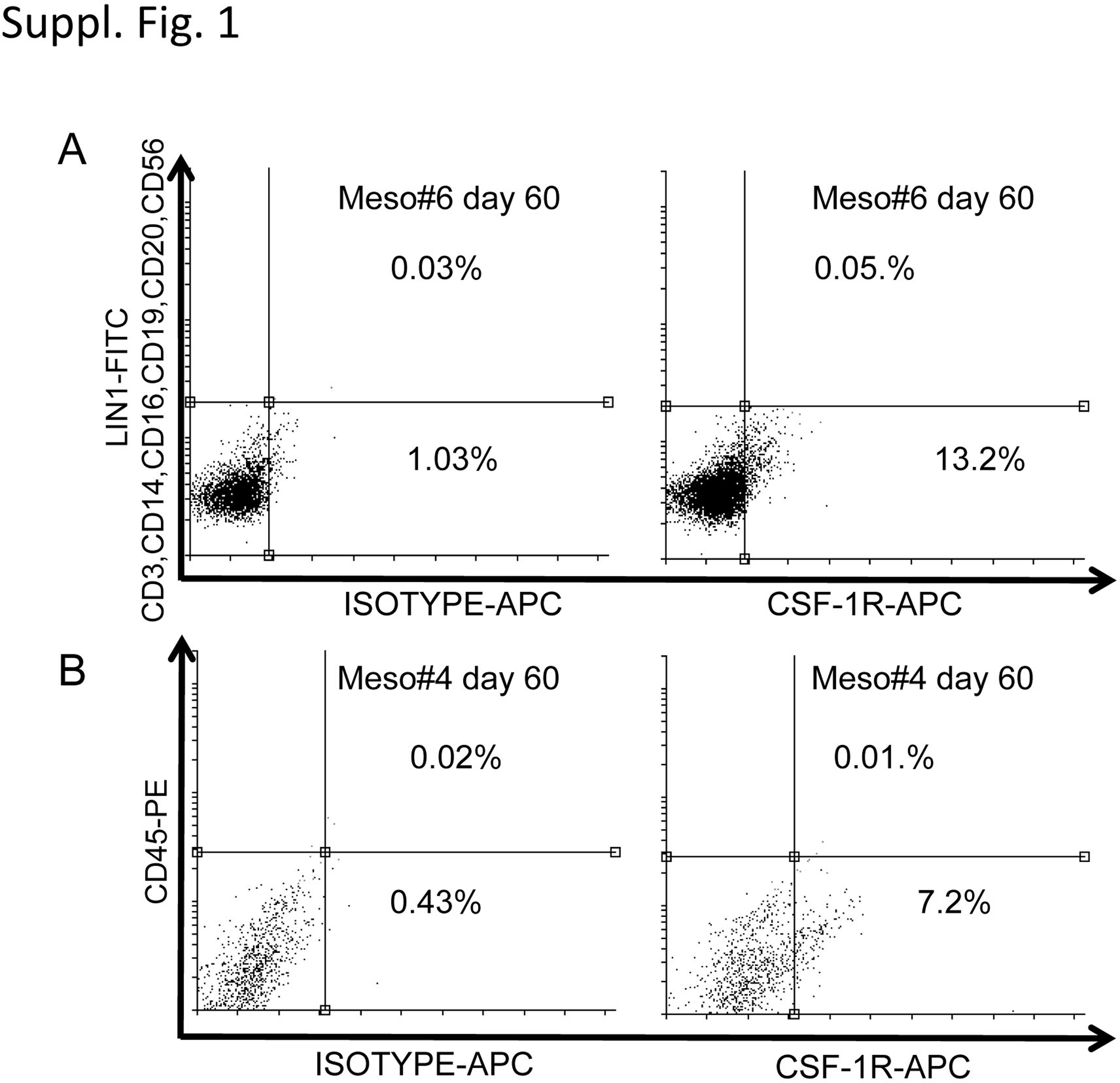

Supplement: Supplementary Figure 1 [file cddis2014136x1.tif]

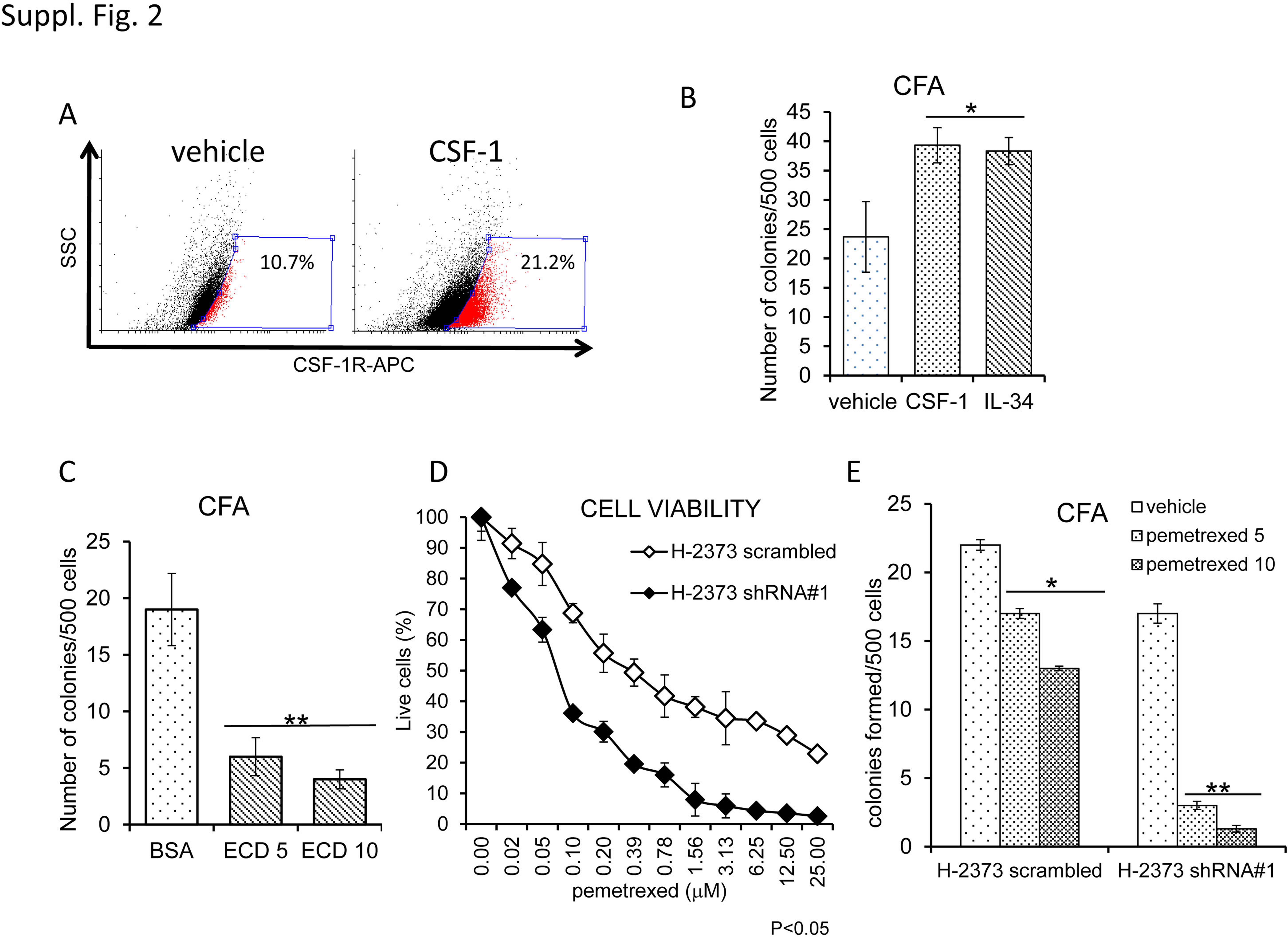

Supplement: Supplementary Figure 2 [file cddis2014136x2.tif]

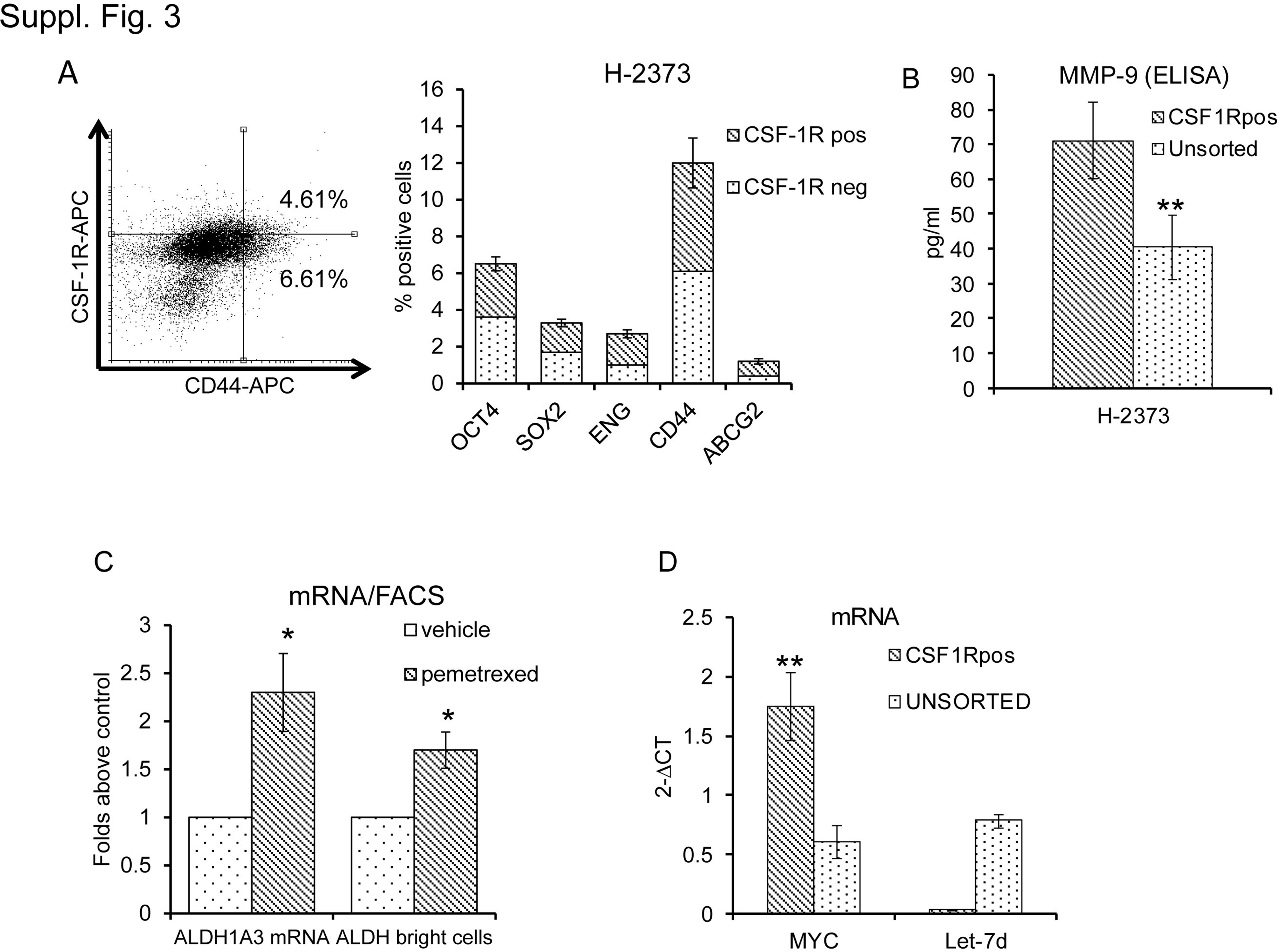

Supplement: Supplementary Figure 3 [file cddis2014136x3.tif]

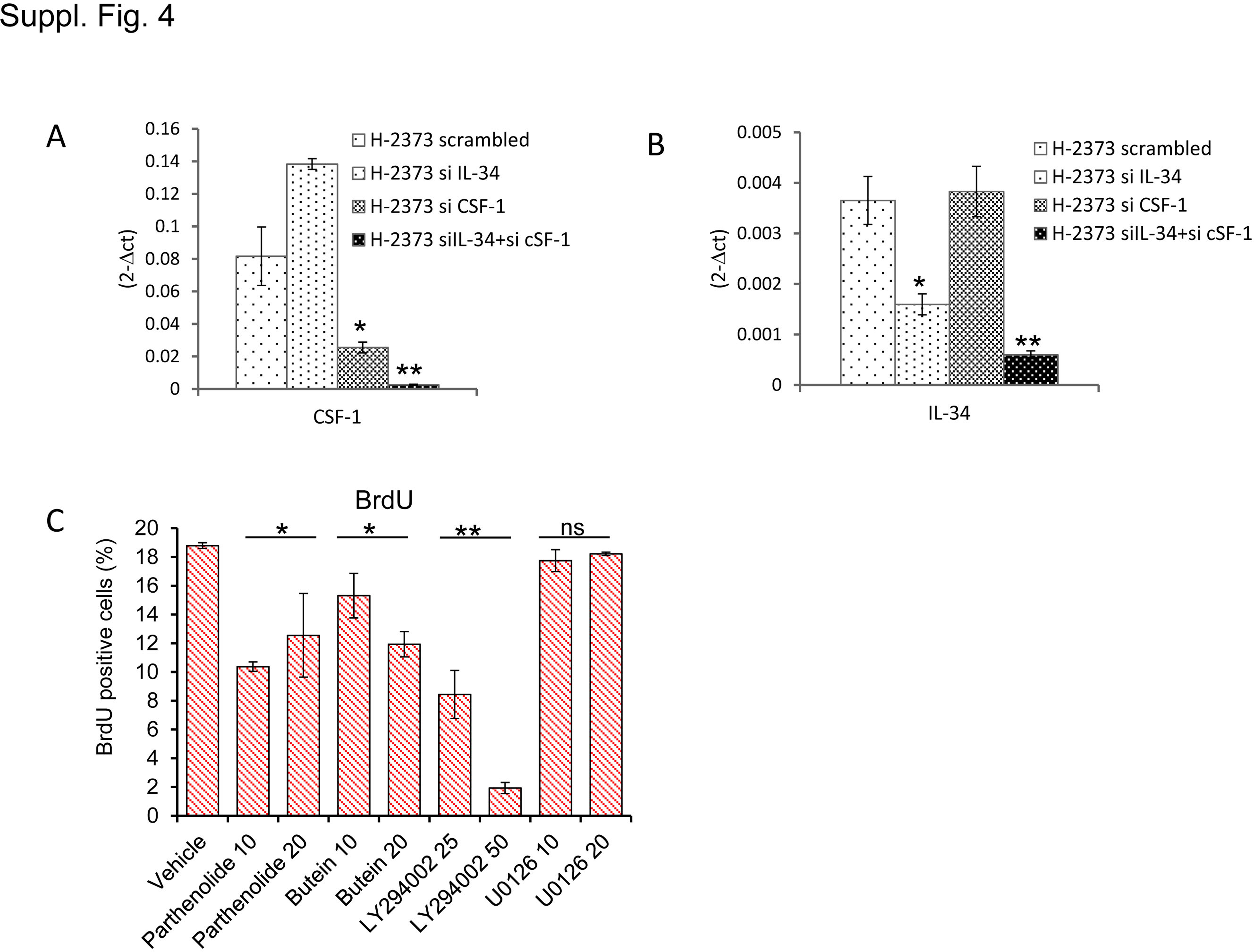

Supplement: Supplementary Figure 4 [file cddis2014136x4.tif]

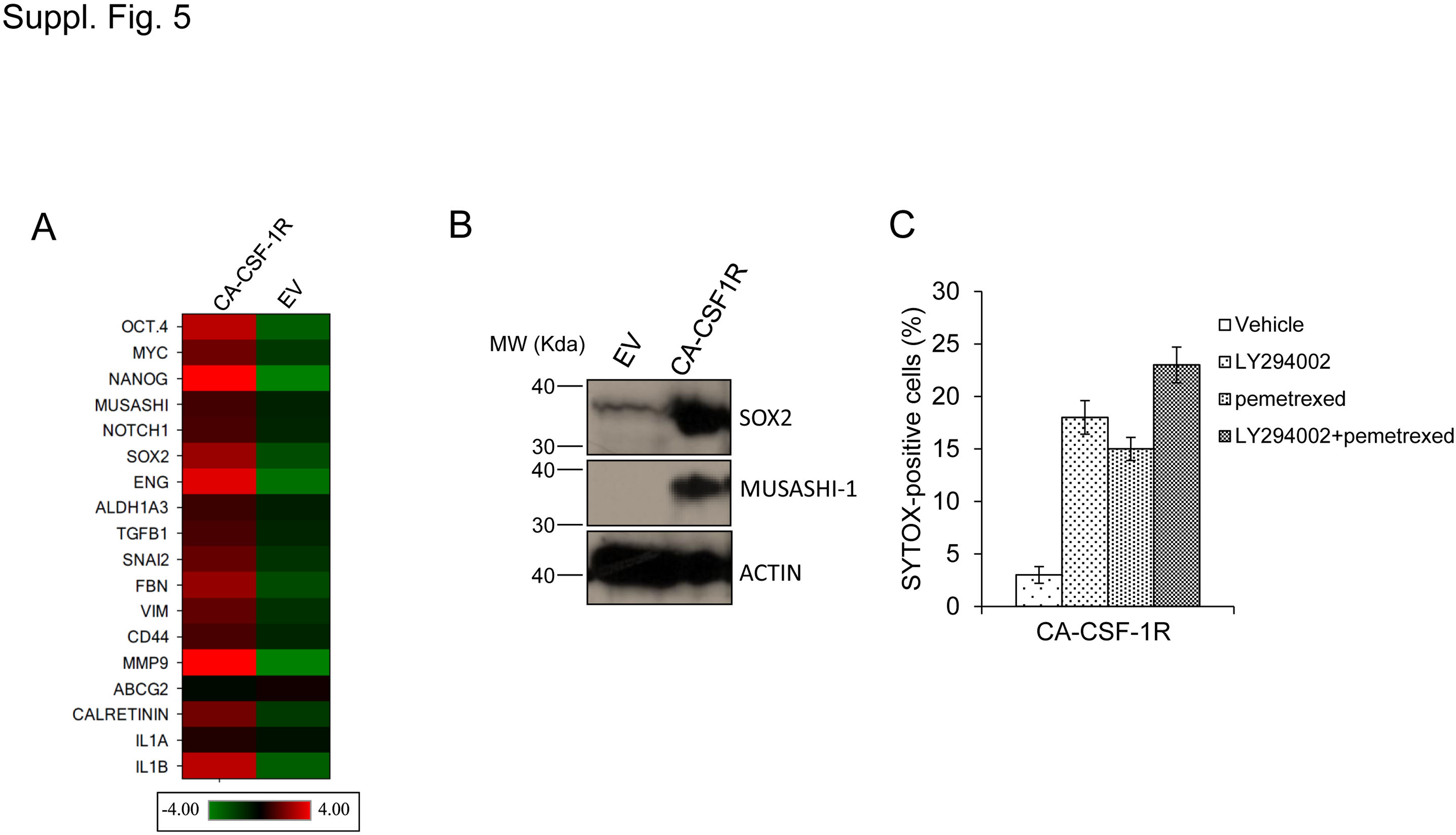

Supplement: Supplementary Figure 5 [file cddis2014136x5.tif]

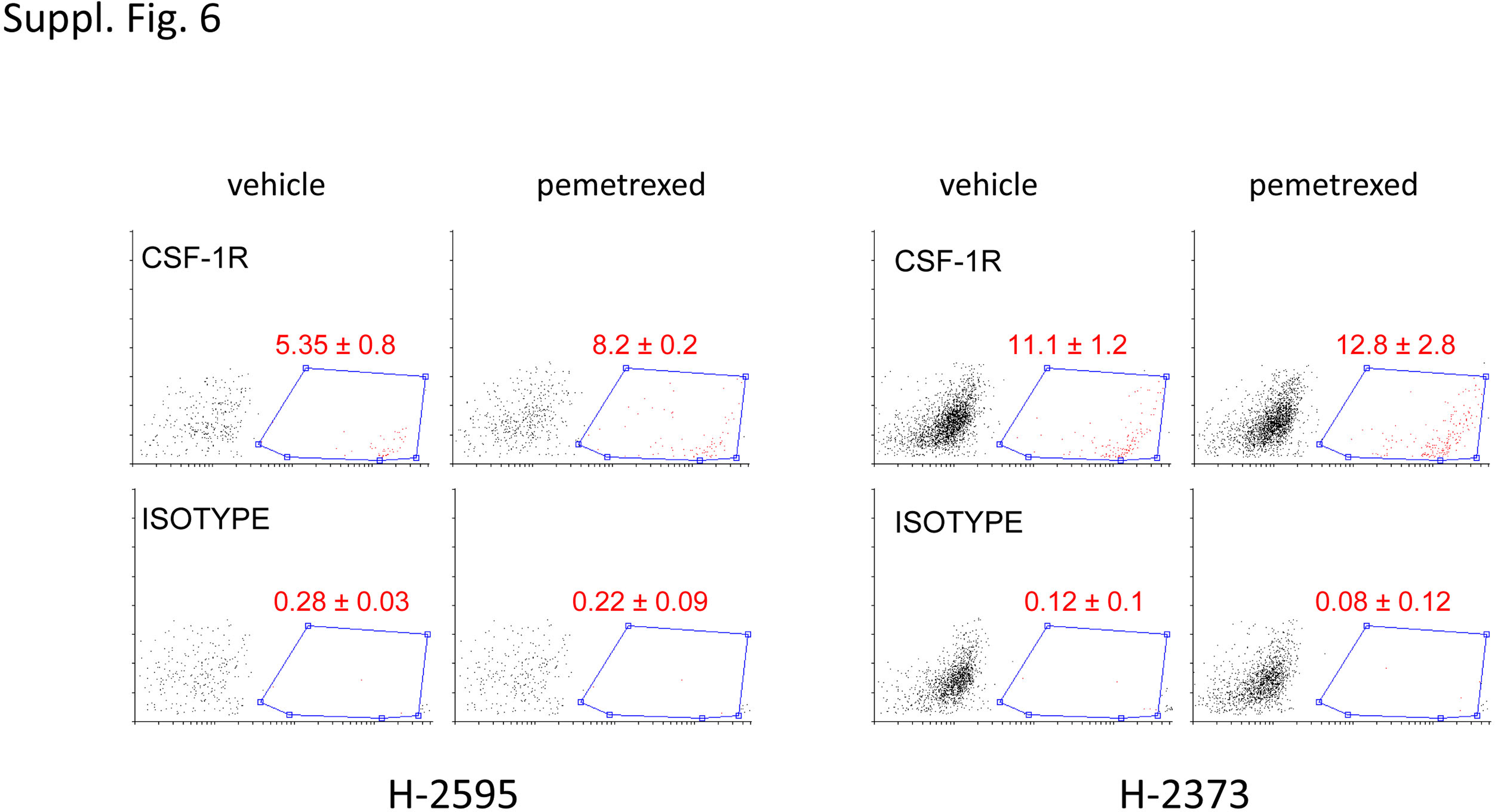

Supplement: Supplementary Figure 6 [file cddis2014136x6.tif]
