# Supplementary material for: Autocrine CSF-1R signaling drives mesothelioma chemoresistance via AKT activation
Source: Cell Death Dis. 2014 Apr 10;5(4):e1167–. doi: 10.1038/cddis.2014.136 (PMC5424113; doi:10.1038/cddis.2014.136)
Supplement: Supplementary Figure Legends and Tables [file cddis2014136x7.doc]

**SUPPLEMENTARY FIGURES AND TABLES**

**Suppl. Fig. 1. Mesothelial origin of the primary CSF-1Rpos cells.** Left. Representative FACS dot plots of primary mesothelioma cultures stained at day 60 after seeding with LIN1 mix (CD3,CD14,CD16,CD19,CD20,CD56) and CSF-1R antibodies (A) or with CD45 and CSF-1R antibodies (B), respectively. Right panels: isotype stained antibodies used to calculate background staining**.** Please note that identical results were obtained for all the primary mesothelioma cell cultures analyzed (n=7).

**Suppl. Fig. 2. A.** **CSF-1 treatment increases the percentage of CSF-1Rpos cells.** Representative FACS dot plots of H-2373 cells treated with vehicle or CSF-1 (25ng/ml) and stained with anti-CSF-1R antibody(right) 48hrs later. Very similar results were obtained when treating the same cells with IL-34 (25ng/ml). **B**. **Clonogenic assay.** H-2373 cells were exposed to vehicle, CSF-1 and Il-34 for 16hrs at the indicated doses and then seeded at clonal density in 6-well tissue culture dishes. Values indicate mean values ± S.E.M. **C. Interference with CSF-1R signaling reduces clonogenicity and resistance to pemetrexed.** Clonogenicity of H-2373 cells exposed to BSA (control) and to increasing concentrations of a CSF-1R decoy (ECD) consisting of the extracellular fragment of the CSF-1R receptor and capable of binding both CSF-1 and IL-34. **D.** Viability assay. H-2373 cells, expressing a scrambled- or two CSF-1R-targeting shRNAs, were treated with pemetrexed at the indicated doses and viable cells counted by the Trypan Blue exclusion assay at 96 hrs. **E.** Clonogenic assay. The same cells as from E were pulse-treated (16hrs) with two increasing dosages of pemetrexed before being seeded at clonal densities. Histograms showing the average colony count at 9 days after seeding. Values indicate mean values ± S.E.M of at least duplicate experiments. *P < 0.05; **P < 0.01 versus the corresponding controls.

**Suppl. Fig. 3. CSF-1Rpos H-2373 cells are enriched for pluripotency, EMT, chemoresistance factors.** **A.** Left. Representative FACS dot plots of H-2373 cells double stained for CD44 and CSF-1R, respectively. Histograms showing the relative percentages of the CSF-1Rpositive and CSF-1R negative cells within the OCT4, SOX2,ENG,CD44 and ABCG2-positive cell subpopulations, as assessed by FACS analysis of double stained, unsorted cells. Bars indicate mean values ± S.E.M of triplicate experiments. **B. CSF-1Rpos cells exhibit high MMP9 levels.** Histograms showing the levels of MMP-9 protein in the conditioned medium (3 days after sorting) of the indicated cell populations, as detected by ELISA. **C. CSF-1Rpos cells exhibit inducible ALDH1A3 mRNA and ALDH activity.** Histograms showing the relative levels of ALDH1A3 mRNA and the percentage of ALDHbright cells in CSF-1Rpos cells treated with vehicle (white bars) or pemetrexed (grey bars), assessed by RTq-PCR and FACS, respectively.  **D. CSF-1Rpos cells exhibit high c-MYC mRNA and low Let-7d microRNA levels.** Histograms showing the average normalized intensity values of c-MYC mRNA and Let-7d microRNA in CSF-1Rpos cells as compared to their unsorted counterparts, as assessed by quantitative PCR. Bars indicate mean values ± S.E.M of triplicate experiments. *P < 0.05; **P < 0.01 versus the corresponding controls (unsorted cells).

**Suppl. Fig. 4**. **A-B**. Quantitative PCR. Analysis of the CSF-1 and IL34 mRNA levels in H-2373 cells transfected with scrambled-, CSF-1siRNA and IL-34siRNA, alone or in combination, and harvested 48hrs later. Data expressed as absolute intensity values normalized for an housekeeping gene (PPIA). Identical results were obtained in H-2595 cells. **C**. **STAT3, NFKB and AKT inhibitors affect the growth of CSF-1Rpos cells.** Histograms showing the percentage of Brdupos cells in purified H-2373 CSF-1Rpos cells treated with the indicated compounds for 48hrs. Bars indicate mean values ± S.E.M of duplicate experiments.

**Suppl. Fig. 5. A. CSF-1R activation drives the expression of pluripotency markers and EMT genes.** Heat map illustrating the (mRNA) levels of the indicated factors in CA-CSF-1R as opposed to EV-LP9 cells as assessed by quantitative PCR (average log2 of the absolute intensity values of duplicate experiments has been used for the heat map). Please note that forced activation of CSF-1R in untransformed cells elicits very similar gene expression changes to the ones observed in Fig. 3A. **B.** Representative Western Blotting of whole cell lysates from logarithmically growing CA-CSF-1R and EV-LP9 cells. Staining with the indicated antibodies indicated high enrichment of the CA-CSF-1R LP9 cells for MUSASHI-1 and SOX2. ACTIN was used as internal control. **C.** Histograms showing the percentage of SYTOX® Dead Cell Stain-labeled CSF-1Rpos cells treated as indicated. The percentage of apoptotic fluorescent cells from two independent experiments is reported.

**Suppl. Fig. 6**. Representative dots plots of the FACS data shown in Fig. 2B with indication of the isotype matched antibody-stained samples.

**Suppl. Table 1.** List of the primers used in this study for the quantitative PCR assays.

| CSF-1R forward  CSF-1R reverse | GAATGACTCCAACTACATTGTC  GTGTAGACAGTCAAAGATG |
| --- | --- |
| CSF-1 forward  CSF-1 reverse | GCTGTTGTTGGTCTGTCTC  CATGCTCTTCATAATCCTTG |
| IL-34 forward  IL 34 reverse | AAACAAAGCTCCGTCCTAAACTG  GCCGCATACTGCAATGAGG |
| OCT.4 forward  OCT.4 reverse | GGGAGATTGATAACTGGTGTGTT  GTGTATATCCCAGGGTGATCCTC |
| SOX2 forward  SOX2 reverse | TACAGCATGTCCTACTCGCAG  GAGGAAGAGGTAACCACAGGG |
| ENDOGLIN forward  ENDOGLIN reverse | TGCACTTGGCCTACAATTCCA  AGCTGCCCACTCAAGGATCT |
| NANOG forward  NANOG reverse | TTTGTGGGCCTGAAGAAAACT  AGGGCTGTCCTGAATAAGCAG |
| MUSASHI forward  MUSASHI reverse | T TCTTCTTCGTTCGAGTCACCA  AAAGTGCTGGCGCAATCG |
| IL1Alpha forward  IL1Alpha reverse | AGATGCCTGAGATACCCAAAACC  CCAAGCACACCCAGTAGTCT |
| IL1Beta forward  IL1Beta reverse | TTCGACACATGGGATAACGAGG  TTTTTGCTGTGAGTCCCGGAG |
| CALRETININ forward  CALRETININ reverse | AGCGCCGAGTTTATGGAGG  TGGTTTGGGTGTATTCCTGGA |
| C-MYC forward  C-MYC reverse | GTCAAGAGGCGAACACACAAC  TTGGACGGACAGGATGTATGC |
| VIMENTIN forward  VIMENTIN reverse | AGTCCACTGAGTACCGGAGAC  CATTTCACGCATCTGGCGTTC |
| ABCG2 forward  ABCG2 reverse | ACGAACGGATTAACAGGGTCA  CTCCAGACACACCACGGAT |
| SNAI1 forward  SNAI1 reverse | TCGGAAGCCTAACTACAGCGA  AGATGAGCATTGGCAGCGAG |
| SNAI2 forward  SNAI2 reverse | CGAACTGGACACACATACAGTG  CTGAGGATCTCTGGTTGTGGT |
| FIBRONECTIN forward  FIBRONECTIN reverse | GAGTTGTCGTGGTCCCTCAG  TGGAGGCGGCATCATAGTTG |
| ALDH1A3 forward  ALDH1A3 reverse | TCTCGACAAAGCCCTGAAGT  TATTCGGCCAAAGCGTATTC |
| NOTCH1 forward  NOTCH1 reverse | TGGACCAGATTGGGGAGTTC  GCACACTCGTCTGTGTTGAC |
| MMP9 forward  MMP9 reverse | TGTACCGCTATGGTTACACTCG  GGCAGGGACAGTTGCTTCT |
| CD44 forward  CD44 reverse | AAGGTGGAGCAAACACAACC  AACTGCAATGCAAACTGCAAG |
| TGFB1 forward  TGFB1 reverse | TGTAAGTGAACATTCAGGTG  TTCCAATAGTCAGCTAAGGA |
| P21 forward  P21 reverse | TGTCCGTCAGAACCCATGC  AAAGTCGAAGTTCCATCGCTC |
| rRNA 18s forward  rRNA 18s reverse | CGGCTACCACATCCAAGGAA  GCTGGAATTACCGCGGCT |
| PPIA forward  PPIA reverse | TCTGAGCACTGGAGAGAAAGG  GGAAAACATGGAACCCAAAGG |

**Suppl. Table 2.** List of the antibodies used in this studies. When used for flow cytometry, the isotype matched or the control antibody used is displayed on the right column.

| phospho-AKT (ser473) | Cell Signaling | Rabbit IgG |
| --- | --- | --- |
| phospho-AKT (Thr308) | Cell Signaling | Rabbit IgG |
|
| phospho-c-Raf (Ser259) | Cell Signaling | Rabbit IgG |
|
| phospho-PDK1 (Ser241) | Cell Signaling | Rabbit IgG |
|
| phospho-GSK-3β (Ser9) | Cell Signaling | Rabbit IgG |
|
| phospho-NFkB (ser563) | Cell Signaling | Rabbit IgG |
| phospho-M-CSF receptor (Tyr723) | Cell Signaling | Rabbit IgG |
| phospho-STAT3(Tyr705) | Santa Cruz biotech | Mouse IgG2b |
| phospho-ERK(Tyr 204) | Santa Cruz biotech | Mouse IgG2a |
| AKT (pan) | Cell Signaling | Rabbit IgG |
|
| STAT3 (pan) | Santa Cruz biotech | Rabbit IgG |
|
| ERK (pan) | Santa Cruz biotech | Rabbit IgG |
|
| NFKB (pan) | Santa Cruz biotech | Rabbit IgG |
|
| OCT4 | Invitrogen | Rabbit IgG |
| SOX2 (D6D9) | Cell Signaling | Rabbit IgG |
| MUSASHI-1 | Cell Signaling | Rabbit IgG |
| CSF1R (C20) | Santa Cruz biotech | Rabbit IgG |
|
| CSF1R (D8) | Santa Cruz biotech | Mouse |
| β-CATENIN | Cell Signaling | Rabbit IgG |
|
| c-MYC | Cell Signaling | Rabbit IgG |
|
| P21 | Santa Cruz biotech | Mouse |
|
| IL1- β | ABCAM | Rabbit IgG |
|
| ACTIN | Santa Cruz biotech | Goat |
| NUCLEOLIN | Cell Signaling | Rabbit IgG |
|
| CALRETININ | ABCAM | Rabbit IgG |
| CD105 PE | Biolegend | PE Mouse IgG1, κ Isotype Ctrl (FC) |
| CD115 APC | Biolegend | [APC Rat IgG1, κ Isotype Ctrl](http://www.biolegend.com/apc-rat-igg1-kappa-isotype-control-1826.html) |
| CD115 PE | Biolegend | [PE Rat IgG1, κ Isotype Ctrl](http://www.biolegend.com/pe-rat-igg1-kappa-isotype-control-1830.html) |
| CD338 PE | eBioscence | PE Mouse IgG2b, κ Isotype Ctrl |
| CD44v6 APC | R&D | Mouse IgG1 APC Isotype Control (Clone 11711) |
| LIN1 (CD3,14,16,19,20,56) | BD Bioscience | Mouse IgG1-FITC |
| CD45 | Ebioscience | Mouse-IgG1-PE |
| Goat Anti-Rabbit DyLight488 | ABCAM |  |
